# Guselkumab shows high efficacy and maintenance in the improvement of response until week 48, a real‐life study

**DOI:** 10.1111/dth.15670

**Published:** 2022-08-08

**Authors:** Luca Mastorino, Niccolò Siliquini, Gianluca Avallone, Mattia Zenone, Michela Ortoncelli, Pietro Quaglino, Paolo Dapavo, Simone Ribero

**Affiliations:** ^1^ Dermatologic Clinic, Department of Medical Sciences University of Turin Turin Italy

**Keywords:** bio‐naïve, guselkumab, psoriasis, real‐life

## Abstract

Guselkumab is an IL‐23 inhibitor that has been demonstrated to be effective and safe for the treatment of moderate‐to‐severe plaque psoriasis in clinical trials. The data pool relating to the use of guselkumab in a real‐life setting is still lacking. To evaluate the efficacy and safety of guselkumab in a real‐life setting, focusing on predictors of early clinical response, a single‐center prospective study was conducted enrolling patients with moderate‐to‐severe psoriasis. The clinical data relating to the efficacy and safety of the drug were acquired at initiation of treatment and at all subsequent clinical follow‐ups: the primary endpoint was PASI90 and PASI100 response at week 12, 24, and 48. Out of the total cohort of 74 patients, 62 (83.8) reached a 48‐week follow‐up 64 (87.8%) reached a 24‐week follow‐up, while 72 (97.3%) a 12‐week follow‐up. Treatment with guselkumab reduced the mean PASI from the initial 11 ± 6.3 to 2.5 ± 3.1 at 12 weeks, to 1.2 ± 1.8 at 24 weeks, and to 0.8 ± 1.6 at 48 weeks. At week 12, a PASI 90 and PASI 100 response was achieved by 44.4% and 23.6% of patients, respectively. After 24 weeks, 63% of patients reported a PASI 90 while 46.1% achieved PASI 100. Previous treatment with one or more other biologics did not impact significantly on the achievement of the PASI 90 and 100 at any endpoints analyzed. We reported no difference between bio‐naïve and non‐naïve patients in the response to guselkumab, high safety, and efficacy was showed in both populations.

## 1 INTRODUCTION

The tumor necrosis factor α (TNFα)–interleukin 23(IL‐23)‐IL17 axis is the basis of the pathophysiology of psoriasis. Anti‐IL‐23 drugs, the most recently introduced group, act upstream of the inflammatory cascade, where this interleukin stimulates and maintains the activation of T helper 17 (Th17) cells whose products (IL‐17 and IL‐22) create a feedforward mechanism.^1^

Guselkumab is the first biologic drug available that specifically inhibits IL‐23 downstream intercellular signaling by binding to its p19 subunit; it is approved for patients with moderate to severe plaque psoriasis who are candidates for systemic therapy. The safety and efficacy of guselkumab were demonstrated by two randomized, double‐blinded, placebo‐controlled, comparator‐controlled, phase III clinical trials: VOYAGE 1 and VOYAGE 2.^2^, ^3^ The NAVIGATE study demonstrated the efficacy of guselkumab in patients who had inadequately responded to ustekinumab.^4^

Real‐life studies are useful in everyday practice as they deal with more complex patients, compared to clinical trials, characterized by multiple comorbidities, multiple therapies, previous failures of biotechnological treatments.

## 2 MATERIALS AND METHODS

Patients with moderate‐to‐severe psoriasis afferent to our center were prospectively recruited between December 2019 and August 2021. The inclusion criterion was the absence of response or contraindication or intolerance to traditional systemic therapies and/or ineffectiveness with previous biologic therapy (anti‐TNFa, anti‐IL17, or anti‐IL12/23). Patients were treated with the standard dose of guselkumab: 100 mg administered by subcutaneous injection at week 0 and week 4, followed by a maintenance dose every 8 weeks.

Patient demographic data, comorbidities, disease characteristics, and previous treatments were collected. Clinical data relating to drug efficacy and safety were acquired at the start of the treatment and in subsequent clinical follow‐up visits. The primary endpoint was the 90% and 100% reduction in the Psoriasis Area and Severity Index (PASI) from baseline (PASI90 and PASI100) at week 12, week 24, and week 48.

All patients gave written informed consent.

Demographic and clinical data were included in the analysis. The student *t* test was used to compare means between normal continuous variables; Mann–Whitney‐*U* test was used for non‐normal distribution. Categorical values were compared with the chi‐square test and fisher's exact test if needed.

All statistical tests were on two sides: *p*‐values <0.05 were considered significant; statistical analyses were performed using Stata/SE12.0 Statistical Software (STATA, College Station, TX).

## 3 RESULTS

Seventy‐five patients were enrolled (Table 1), 43 (58%) of whom were male. The mean age of the patients at the start of therapy with guselkumab was 46.6 ± 17. The mean body mass index (BMI) was 24.2 with a slight difference between the two sexes (23 for women and 25 for men, *p* = 0.08). Guselkumab was the first biologic treatment for psoriasis (bio‐naïve patients) in 43 patients (58.1%). Cardiovascular diseases, obesity, and cancer were the most represented comorbidities in the series (25.6%, 13.5%, and 9.5%, respectively).

**TABLE 1:** Baseline characteristics of the study population

Clinical features	*N* = 74
**General characteristics**	
Male sex, *n* (%)	43 (58)
Age (mean)	46.6 ± 17
BMI (mean)	24.2 ± 4.9
PASI score prior guselkumab (mean)	11 ± 6.4
**Biologic therapies, *n* (%)**	
Bio‐naive	43 (58.1)
Previous biologic therapy	31 (41.9)
**Comorbidities, *n* (%)**	
Cardiovascular (hypertension, heart failure, coronary syndrome)	19 (25.6)
Obesity	10 (13.5)
Dyslipidemia	5 (6.7)
Diabetes	6 (8.1)
Cancer	7 (9.5)
Infections	3 (4)

Abbreviations: BMI, body mass index; PASI, psoriasis area and severity index.

Considering the clinical features of psoriasis at baseline, the most involved body site was the scalp (56 patients, 75.6%, 22 of whom also had facial involvement); 11 (14.9%) patients presented psoriatic onychopathy and 14 (18.9%) also showed palmoplantar involvement. This site was the only affected site in 4 (5.4%) patients.

Out of the total cohort of 74 patients, 62 (83.8) reached a 48‐week follow‐up 64 (87.8%) reached a 24‐week follow‐up, while 72 (97.3%) a 12‐week follow‐up.

**FIGURE 1:**
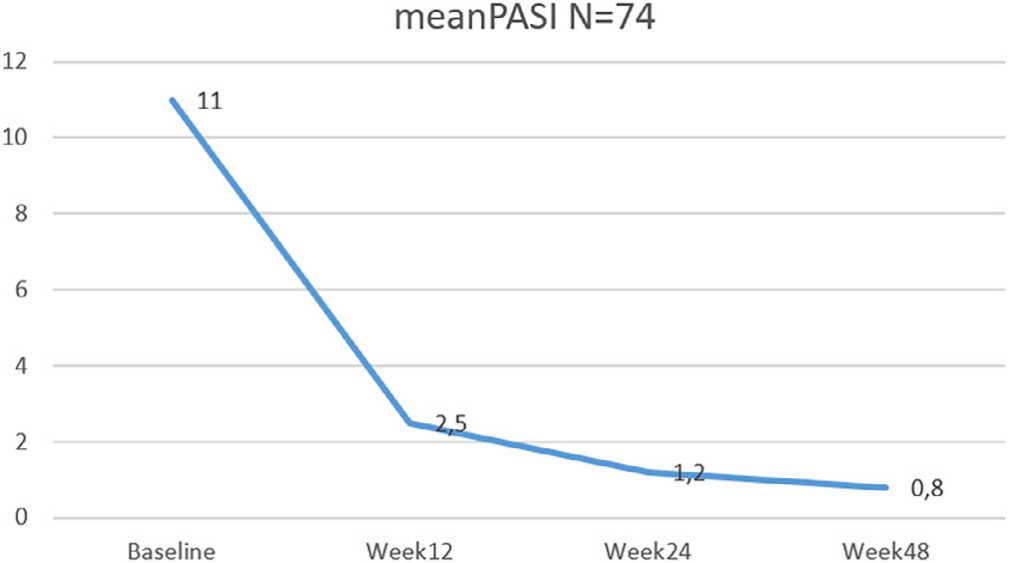
Mean PASI at weeks 12, 24, 48

**FIGURE 2:**
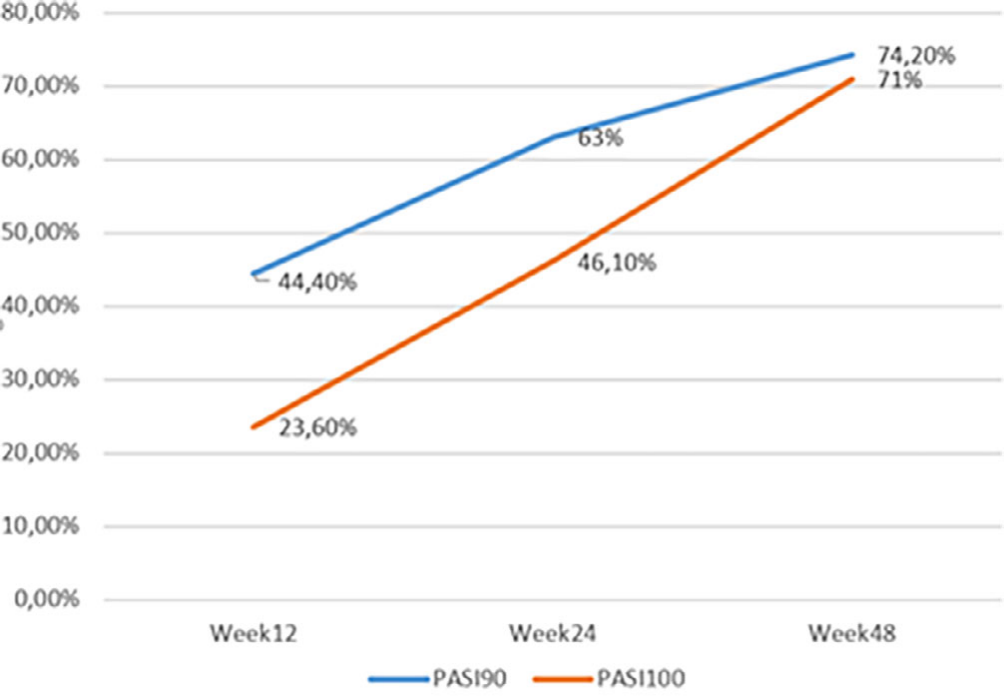
Percentage of patients reaching PASI 90 and PASI 100 at weeks 12, 24, and 48

From the initial mean PASI of 11 ± 6.3, the treatment with guselkumab was effective in reducing the PASI to 2.5 ± 3.1 at 12 weeks, to 1.2 ± 1.8 at 24 weeks, and to 0.8 ± 1.6 at 48 weeks (Figure 1). At week 12, a PASI90 and PASI100 response were achieved by 44.4% and 23.6% of patients, respectively. After 24 weeks, 63% of patients reported a PASI90 while 46.1% achieved PASI100. After 48 weeks 74.2% of patients reported a PASI 90 while 71% achieved PASI100 (Figure 2).

There was no statistically significant association between age, sex, baseline PASI or disease duration and achievement of PASI90 at 12 weeks. Comorbidities did not affect the clinical response in any of the included patients.

**FIGURE 3:**
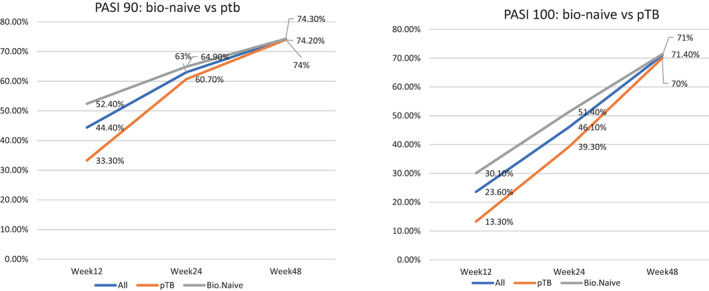
Comparison of PASI between Bio‐Naïve patients and Bio‐experienced patients

At week 12, 52.4% of bio‐naïve patients achieved PASI90 and 30.1% of them achieved PASI100 response, while among non‐bio‐naïve patients only 33.3% and 13.3% of patients achieved these responses, respectively (*p* = 0.11 and *p* = 0.08). After 24 weeks, a PASI90 and PASI100 response were observed for 64.9% and 51.4% of bio‐naïve patients respectively, while the same response was achieved respectively by only 60.7% and 39.3% of patients if considering only patients with previous biologic therapy (*p* = 0.73 and *p* = 0.33). At 48 weeks of follow‐up, a PASI90 and PASI100 response were observed in 74.3% and 71.4% of bio‐naïve patients respectively, while the same response was achieved respectively by 74% and 70.1% of patients with previous biologic therapy (*p* = 0.9 and *p* = 0.6) (Figure 3).

Within the subpopulation of patients with exclusive involvement of difficult body areas (4 patients with exclusive palmoplantar involvement), one patient reached PASI 90 and 100 at weeks 24 and 48, and another patient reached PASI 90 at the same time points.

Six (8%) patients reported mild adverse events; 3 (4%) cases of injection site reaction; 2 (2.7%) cases of asthenia; 1 (1.3%) case of headache. No serious adverse event was reported. Seven patients (9.3%) interrupted the treatment after a mean time of 31.1 weeks (*SD* 12.75), two patients due to secondary inefficacy and five due to primary inefficacy.

## 4 DISCUSSION

We report a large prospectively collected cohort of patients treated in a real‐life setting with guselkumab. Patients reached good achievement of disease control in terms of PASI90 and PASI100, confirming what has been reported by other real‐life studies.^5^, ^6^, ^7^, ^8^, ^9^, ^10^, ^11^, ^12^, ^13^, ^14^, ^15^

The mean age of the population, 46.6 ± 17 years, was comparable to the one reported by clinical trials and other monocentric real‐life series^6^, ^7^, ^8^, ^11^, ^12^ The mean PASI at the start of treatment (11 ± 6.3) corresponds to a halved value compared to VOYAGE studies^2^, ^3^ and is slightly lower than the value reported by other real‐life studies.^6^, ^7^, ^8^, ^11^ As regards the clinical response at week 12, we found a substantial overlap with the one reported by Megna et al.,^6^ whose study population had an initial mean PASI about four points greater but a lower percentage of bio‐naïve patients (35% compared with our 58.1%) compared to ours. Galluzzo et al.^5^ reported a lower PASI90 (36% vs. our 44.4%) and a lower PASI100 (18% vs. our 23.6%) at 12 weeks, despite the significantly higher initial mean PASI (20 ± 13.3). The pivotal studies^2^, ^3^ and other real‐life case series^8^, ^9^ instead report a first follow‐up at 16 weeks, a data not comparable with ours, especially considering the additional dose of drug administered in the time between the 12th and 16th week.

Considering the 24 weeks follow‐up, we observed a PASI90 and a PASI100 of 63% and 43.1%, respectively: these data were lower than those of the VOYAGE studies, whose populations were characterized by a greater presence of bio‐naive patients and a definitely higher mean starting PASI^2^, ^3^ Snast et al.^7^ described a population which was different from the other series reported in the literature, having included only multi‐failure patients (mean starting PASI 14 ± 6): the PASI90 reported at 24 weeks was similar to ours (62% vs. 63%, respectively), while the PASI100 was lower (17% vs. 43.1% in our series). PASI90 and PASI100 at 28 weeks reported by Megna et al.^6^ were 69.6% and 39.1%, respectively, while those observed by Galluzzo et al.^5^ were 72.5% and 55%, respectively: these are similar values compared to ours, even if calculated at 28 weeks of treatment.

At 12 months PASI 90 and PASI 100 was reached by 78.9%, and 63.2% of patients in the study of Galluzzo et al., 73.9% and 43.5% of patients reached the same outcomes in the work of Megna et al., this data are similar, if not better, to our values at 48 weeks 74.3% and 71.4% respectively.^5^, ^6^

The safety profile was in line with the pivotal studies and other real‐life studies.^2^, ^3^, ^5^, ^6^, ^7^, ^8^, ^9^, ^10^

Within the subpopulation of patients with exclusive involvement of difficult areas (4 patients with exclusive palmoplantar involvement), two patient did not reach PASI 90 at 24 weeks; the other patients did not reach these outcomes.

Our series confirms the early efficacy and the safety of guselkumab, supporting data derived from VOYAGE 1 and 2,^2^, ^3^ despite differences in patient characteristics such as disease severity at baseline and exposure to prior biological treatments. Guselkumab is also reliable in terms of retention rate: although the observation period of the patients was relatively short, only one patient discontinued the treatment due to ineffectiveness.

Among the real‐life experiences reported in the literature to date, our population study included the highest absolute number of bio‐naïve patients at the start of treatment. Our finding in contrast with what reported by Galluzzo et al.^6^ (better response to guselkumab in patients with a low number of previous therapies, but no valid comparison was possible due to different statistic methods), did not show any significant differences between bio‐naïve and non‐naïve patients as observed in other real‐life experiences.^8^, ^9^ We found a trend for better response in bio‐naïve, despite this did not reach the statistical significance, probably due to the low sample size. Hung et al. have recently described similar effect of biologics exposure to guselkumab response.^14^

Ruggiero et al. showed, in a small cohort of patients, the efficacy of guselkumab even in the population that had previously failed ustekinumab, secukinumab, and ixekizumab, with a reduction in mean PASI similar to ours.^15^

Galluzzo et al.^5^ also found a reduced response depending on the number of comorbidities, while in our series this association was not statistically significant.

To our knowledge, ours is the real‐life study with the largest sample size that assessed response based on previous experience with biological drugs to guselkumab at 48 weeks. Our data confirm the previously reported efficacy and safety parameters with no substantial differences between bio‐naive patients and bio‐experienced patients.

The low sample size is one of the main limitations of our study, partly compensated by the follow‐up up to 48 weeks achieved by the larger part of our population. The retrospective analysis of the data limits the generalizability of the collected data, furthermore, apart from the use of previous biological drugs no analysis of further subgroups, such as joint involvement, smoking habits, nor a specific analysis of difficult sites was performed. Certainly, studies involving a greater number of patients and characterized by a longer follow‐up period are needed to evaluate the efficacy and safety of guselkumab in a real‐life setting.

Newly developed systems that approach the evaluation of differences in therapeutic responses using noninvasive methods such as tape stripping (ref Zhai) could be approached with future studies.^16^

## AUTHOR CONTRIBUTIONS

Luca Mastorino and Niccolò Siliquini performed research work and data analysis, wrote the first draft and performed the revision. Gianluca Avallone and Mattia Zenone performed data research. Michela Ortoncelli, Pietro Quaglino, and Paolo Dapavo supervised the work. Simone Ribero supervised and revised the work and performed data analysis. All authors read and approved the paper.

## CONFLICT OF INTEREST

The authors declare no conflicts of interest.

## Data Availability

Data available upon reasonable request.
